# Exploring the Acceptability of Integrating Dietitians Into Primary Care Dental Services for Children

**DOI:** 10.1111/hex.70753

**Published:** 2026-07-03

**Authors:** Lauren Hallewell, Raul Bescos, Zoe Brookes, Tracey Parkin, Robert Witton, Patricia Casas‐Agustench

**Affiliations:** ^1^ School of Health Professions, Faculty of Health University of Plymouth Plymouth UK; ^2^ Peninsula Dental School, Faculty of Health University of Plymouth Plymouth UK

**Keywords:** children, dentistry, dietitian, interprofessional, nutrition

## Abstract

**Background:**

Dental caries is a common childhood non‐communicable disease strongly associated with high sugar intake. Beyond oral health, a poor diet can affect children's growth, development, and overall well‐being. While dietary advice is central to preventive dental care, some primary care dental clinics have introduced body mass index (BMI) assessments and further dietary advice to address wider nutritional concerns in children. However, these efforts are often constrained by limited nutrition training, time constraints and concerns about sensitivity. Integrating dietitians into primary dental care may help overcome these barriers by enhancing dietary guidance, supporting broader nutritional needs and facilitating structured BMI assessments with appropriate follow‐up.

**Aim:**

To explore the acceptability of integrating dietitians into primary dental care for children, guided by the theoretical framework of acceptability (TFA).

**Methods:**

Dental professionals and caregivers were recruited from South West England, and dietitians from across the United Kingdom (April–December 2024). Semi‐structured interviews were analysed inductively using reflexive thematic analysis. Findings were interpreted through the TFA to examine how emotional, ethical and structural factors shaped perceptions of acceptability.

**Results:**

Semi‐structured interviews were conducted with 23 participants: 10 dental professionals, 6 dietitians and 7 caregivers. Four inductive themes captured participants reasoning: (1) Balancing enthusiasm and sensitivity; (2) Negotiating the realities of integration; (3) Clarifying boundaries and expertise and (4) Building collective confidence. These themes aligned closely with the TFA, showing how emotional, ethical and structural factors interacted. Acceptability emerged as a dynamic balance of emotional and ethical alignment, practical feasibility, conceptual clarity and shared confidence. Perceived effectiveness was a unifying thread, with integration seen as acceptable when safeguards ensured emotional safety, ethical grounding and organisational support.

**Conclusion:**

Integrating dietitians was viewed as widely acceptable when collaboratively, sensitively and structurally delivered and supported.

## Patient or Public Contribution

Caregivers contributed by participating in qualitative interviews and sharing their experiences and perspectives. Their input directly informed the analysis and interpretation of the findings.

## 1 Introduction

Diet is a common risk factor for both oral health and growth in childhood, influencing outcomes from dental caries to obesity. Dental caries is a highly prevalent chronic disease in children, and the leading cause of hospital admissions and missed school days [1]. Evidence also suggests an association between paediatric dental caries and body mass index (BMI), with underweight, overweight or obese children often exhibiting higher caries rates than those of a healthy weight [2, 3]. In paediatric care, BMI is commonly used to assess growth [4] and provide a practical estimate of overweight and obesity when interpreted with age‐and sex‐adjusted charts [5]. However, it does not directly measure adiposity and should be interpreted alongside broader clinical, developmental and social context [5].

Children living with underweight, overweight or obesity may experience increased oral‐disease risk through mechanisms such as micronutrient deficiencies [6], altered oral microbiome [7, 8, 9, 10] or frequent consumption of sugar‐rich foods, which can also arise in the dietary management of underweight status [11, 12, 13, 14]. Recognising these overlaps, some dental clinics have begun to incorporate BMI measurements and brief lifestyle interventions, led by dental teams, as part of a broader public health approach [15, 16, 17, 18, 19, 20, 21, 22]. This shift aligns with National Institute for Health and Care Excellence guidance recommending opportunistic measurements to identify children at risk of excess weight, outside of the National Childhood Measurement Programme (NCMP), which operates as a universal, school‐based programme [5]. Dental settings rely on families who attend services, introducing an element of self‐selection and potential inequities in reach. Such initiatives also remain inconsistent, with limited evidence of long‐term benefit and ongoing concerns regarding workload, stigma and limited referral pathways in dental environments [21]. Dental services therefore represent an opportunistic setting that can complement, but not replicate, population‐level screening programmes.

Most children in the United Kingdom receive dental care in primary care settings from general dental practitioners, dental therapists, dental hygienists or dental nurses [23]. Among this routine dental care, dietary advice is a long‐established component of caries prevention [24]. However, in primary care practice, such advice is often brief, generic and primarily focused on reducing sugar intake, rather than promoting a more holistic approach to nutrition [24, 25]. Dental professionals frequently cite barriers including time constraints, insufficient nutrition training and uncertainty about when and how to refer families for specialist support [25]. In the United Kingdom, dietary advice in dentistry continues to be informed by Delivering Better Oral Health [26]. Meanwhile, caregivers value dietary guidance that is consistent, personalised and non‐judgemental, although some describe current approaches as inconsistent and occasionally insensitive to their circumstances [25].

The introduction of BMI assessment and enhanced dietary counselling for children reflects growing recognition of primary care dental settings as important sites for delivering broader preventive health initiatives [27, 28]. These clinics provide regular, routine points of contact across childhood [29]. Integrating dietitians within primary dental care could build on this opportunity, addressing barriers previously identified by dental professionals and reducing the burden of external referrals, which are often constrained by waiting times, communication breakdowns and fragmented follow‐up [30]. In many primary care contexts, other public health professionals, such as school nurses, health visitors and general practitioners, already play key roles in supporting families with nutrition, growth and oral health promotion [31]. Effective collaboration between primary care dental teams and dietitians within this wider preventive network highlights opportunities for improved coordination, consistency and shared responsibility for childhood health [32, 33]. However, the success of these initiatives depends on both professional capacity and family acceptability.

Integrating dietitians within dental teams has been proposed as a strategy to strengthen preventive care [34, 35]. Dietitians could enhance this emerging model by providing specialist expertise in nutritional assessment, anthropometric measurements and behaviour‐change communication, offering complementary skills to dental teams [36]. Emerging evidence suggests that both dental professionals and adults perceive potential value in such collaboration, particularly when services extend beyond caries prevention to address systemic conditions [37]. In this study, ‘integration’ refers specifically to the co‐location of dietitians within primary dental care settings, meaning that dietitians are physically situated in dental clinics and directly involved in patient care. However, little is known about how such integration within primary dental care for children is perceived by caregivers, dietitians and dental professionals, or how acceptable it would be within routine practice.

Guided by the theoretical framework of acceptability (TFA) [38], a widely used model for assessing how acceptable healthcare interventions are to service users and practitioners and conceptualising acceptability through seven constructs (affective attitude, burden, ethicality, intervention coherence, opportunity costs, perceived effectiveness and self‐efficacy), this qualitative study explored the perceptions of caregivers, dental professionals and dietitians regarding the integration of dietitians into primary dental care for children. For clarity, we use the term ‘children’ to refer to the paediatric population aged 0–18 years, recognising this spans infancy to adolescence. This definition reflects the conceptual scope of the study; however, the empirical sample comprised caregivers of children aged 13 years or younger who were attending services. It examined how such integration, through BMI measurements, follow‐up dietary support and enhanced nutrition‐related oral health advice, might be understood and accepted, thereby informing the development of integrated, patient‐centred preventive care models that address both oral and systemic health needs.

## 2 Methods

### 2.1 Study Design

This study used a qualitative approach with a descriptive research design, appropriate for exploring experiences and perceptions. To ensure transparency, the Consolidated Criteria for Reporting Qualitative Research checklist was utilised [39].

### 2.2 Participants and Sampling

Dental professionals were recruited using purposive sampling through four primary care dental settings where dental students are trained, providing care for children in South West England between April and September 2024. Across the four settings each year approximately 6260 appointments are offered to 2500 children. Recruitment was facilitated via emails containing recruitment posters, as well as posters displayed in staff areas and internal communications. Eligible participants were those providing clinical care to children in primary care, and thus included dental hygienists, dental therapists, dentists and dental nurses.

Caregivers were also recruited using purposive sample from the same dental settings. Recruitment materials, including posters, were placed in waiting areas, and the lead author attended paediatric clinic sessions to distribute study information directly to caregivers. Eligible participants were caregivers of children attending paediatric dental clinic sessions, which typically serve children aged 13 years or younger. Recruitment within this age range reflected service structure and feasibility considerations. Interviews explored broader perceptions of integrated dietetic care rather than age‐specific experiences. Caregivers were able to draw on experiences across age groups, and age ranges of children are therefore reported in aggregated categories.

Dietitians were convenience sampled between September and December 2024 and eligible if they were UK‐based, registered with the Health and Care Professions Council, and working with children. The British Dietetic Association (BDA) Paediatric Specialist Group facilitated recruitment by sharing research posters via social media and their newsletter.

Recruitment posters contained study details and a QR code linking to an expression of interest email to the lead author. Interested participants received a participant information sheet and an online consent form, with opportunities to ask questions before enrolling.

### 2.3 Data Collection

Participants chose their preferred methods of participation: in‐person at the dental setting, via videoconference or over the phone. All participants provided written consent to participate in the study. The study was approved by the University of Plymouth, Faculty of Health Research Ethics and Integrity Board (Reference: 5706, 5479, 4833).

Participants provided optional demographic (e.g., age, gender and ethnicity) and role‐specific data. Dietitians shared this information during interviews; dental professionals described their specialisation and years of experience, and both dental professionals and caregivers completed the remaining demographic questions retrospectively via an online form.

Interviews were designed and structured using the TFA, which examines seven constructs: affective attitude, burden, ethicality, intervention coherence, opportunity costs, perceived effectiveness and self‐efficacy [38]. The TFA informed both the interview guide and subsequent analysis, allowing systematic exploration of participants cognitive and emotional responses.

A semi‐structured format (File S1) was adopted to balance theoretical guidance with flexibility for participants to raise issues of personal importance. Initial questions for all participants explored current practices/experiences related to oral health, dietary assessment and nutrition counselling. For caregivers and dental professionals, a short video/audio resource produced by the BDA [36] introduced the role of a dietitian, supporting informed discussion. Dietitians, in turn, were provided with a summary describing current dental practices related to dietary advice and BMI measurements, ensuring background information was tailored to each group's professional context.

All participants were invited to discuss two hypothetical scenarios depicting a dietitian physically integrated within a dental setting and providing in‐house support (File S1). These scenarios, informed by existing research, illustrated practical examples of dietitian roles for children, including dietary counselling for oral health concerns (regardless of weight) and BMI‐for‐age‐and‐sex measurement with follow‐up support within the same clinic (for all children). This approach encouraged participants to consider the acceptability of integration in real‐world contexts. Integration was conceptualised as co‐location, with referrals referring to internal dental‐clinic pathways rather than external dietetic services. Interviews with dental professionals and dietitians also explored prior experiences of interprofessional collaboration, perceived barriers and potential facilitators to integration. The topic guide was piloted with one representative from each group to ensure clarity and relevance and refined accordingly.

Recruitment continued until data saturation was achieved, defined as the point at which no new themes emerged during data analysis [40]. All interviews were conducted by the lead author, a registered dietitian and doctoral researcher. Interviews were conducted in person, online or by telephone. All interviews were audio‐recorded and transcribed verbatim by the lead author for analysis. No other individuals were present during data collection.

### 2.4 Analysis

Given the exploratory nature of this study, reflexive thematic analysis was conducted following Braun and Clarke's framework [41, 42]. This approach was applied inductively, allowing themes to emerge directly from participants accounts before being considered in relation to the TFA [38].

The anonymised transcripts were analysed using a six‐step process. The analysis began with familiarisation, and initial reflections were noted. Coding using Nvivo 12 [43] was performed by the lead author at both semantic and latent levels, ensuring depth in data interpretation. Next, preliminary themes and subthemes were generated, grouping similar codes and refining them iteratively. To ensure rigor and reflexivity, a collaborative approach was adopted, where two researchers engaged in discussions, refining themes and subthemes while maintaining alignment with the original data. Braun and Clarke's 15‐point checklist for good thematic analysis guided this process, ensuring traceability and verification [44]. Following Braun and Clarke's recommendations, the analysis remained dynamic, involving constant iteration between coding and theme generation. In the final stage, three researchers synthesised the findings, selecting representative quotations for the results section.

Once preliminary themes were generated, they were examined deductively to explore how they aligned, extended, or diverged from the seven constructs for the TFA. This iterative process allowed the TFA to be used as an interpretational lens rather than a coding framework.

### 2.5 Trustworthiness

To enhance confirmability of the findings, a ‘new member‐checking’ approach was adopted, where all participants were invited to review and provide feedback on the study findings rather than their individual transcripts. This allowed them to reflect on how their experiences aligned with those of others, supporting accurate representation of the data [45]. Four participants responded, indicating that the findings resonated with their perspectives and offering brief affirming comments. This approach was preferred over traditional member checking as it enhances participant engagement, provides richer feedback, and minimises the risk of potential re‐traumatisation when discussing the sensitive topic of body weight [45].

Three researchers were involved in the analytical process to ensure confirmability through triangulation. The lead author adopted a reflexive approach, systematically recording reflections throughout data collection and analysis. As a registered dietitian with no prior experience conducting qualitative interviews, the lead author received formal training in methodology and ongoing supervision to ensure rigour. The lead author had no relationship with the participants or the clinic settings. The non‐clinical role helped reduce power imbalances and encourage open dialogues; however, the shared professional background with dietitian participants may have introduced subtle bias. Additional strategies to enhance trustworthiness included the use of semi‐structured interviews, interdisciplinary coding and team‐based review, which supported consistency and strengthened dependability. Thick, rich descriptions and participant quotes were used to provide contextual depth and contribute to understanding the acceptability of integrating dietitians into primary dental care for paediatric populations.

## 3 Results

This study included 23 participants: 10 dental professionals, 6 dietitians, and 7 caregivers. Interviews lasted 28–61 min (mean 42 min), with average durations of 45 min for dietitians, 42 min for dental professionals, and 35 min for caregivers. To protect participant confidentiality and prevent deductive disclosure, demographic data were reported in aggregate rather than linked to individual responses [46]. Demographic breakdowns are available in Tables S1–S3.

Ten dental professionals with experience ranging from 12 to 34 years (mean 20.5 years) participated, including general dental practitioners (*n* = 3), dental nurses (*n* = 3), dental hygienists (*n* = 2) and dental therapists (*n* = 2) all working in primary care. Seven provided additional demographic data, of whom six identified as female and one as a male. Most participants reported working across multiple settings, including private practice, National Health Service (NHS) dentistry and academia; none selected working in secondary dental care. Six female dietitians provided demographic data, with paediatric experience ranging from 1 to 36 years, averaging 20 years.

Five caregivers provided demographic data. Four identified as mothers and one as a father, with children aged between 3 and 14 years. Educational backgrounds varied from secondary school to postgraduate degrees. Employment status included full‐time (*n* = 2), part‐time (*n* = 1), self‐employed (*n* = 1) and homemaker (*n* = 1).

### 3.1 Observed Themes

Initial inductive coding generated four higher‐order themes that reflected participants experiences and reasoning around integrating dietitians into primary dental care for children [1]: balancing enthusiasm and sensitivity [2], negotiating the realities of integration [3], clarifying boundaries and expertise and [4] building collective confidence. These themes were subsequently examined through the seven constructs of the TFA, enabling a structured interpretation of how emotional, ethical and practical factors influenced perceived acceptability. Table 1 summarises the four inductive themes, their alignment with specific TFA constructs and the analytical linkages that informed interpretation.

**Table 1:** Inductive themes mapped to constructs of the theoretical framework of acceptability (TFA).

Inductive theme	Summary of what the theme captures	Mapped TFA constructs	Analytical linkage
Balancing enthusiasm and sensitivity	Participants expressed optimism about integrating dietitians into dental care for diet‐related oral health advice, but this coexisted with emotional discomfort around weight‐related discussions. Essential safeguards were suggested to ensure sensitive handling.	*Affective attitude, ethicality, perceived effectiveness*	Reflects positive and negative emotional responses (*affective attitude*), combined with moral and emotional tension about weight‐focused dialogue (*ethicality*). Highlights how emotional and ethical dimensions jointly shape perceived acceptability.
Negotiating the realities of integration	Participants recognised both logistical and motivational demands of integration, including new workloads for professionals and engagement challenges for caregivers.	*Burden, opportunity costs, perceived effectiveness*	Captures perceptions of effort, workload and *opportunity costs* required to engage with or deliver the intervention. Demonstrates how participants weighed immediate inconvenience against perceived long‐term preventive value.
Clarifying boundaries and expertise	Participants reflected on existing collaborative practices, highlighting how dental and dietetic roles both align and diverge, while valuing the recognition of dietitians as nutrition experts.	*Intervention coherence, ethicality, perceived effectiveness*	Describes how participants understood the purpose and function of integration. *Ethicality* linked to professional boundaries and concerns about role legitimacy and scope.
Building collective confidence	Professional confidence and readiness for interprofessional working were seen to depend on structural and resource support, standardised protocols, training and ongoing coordination.	*Self‐Efficacy, intervention coherence, perceived effectiveness*	Reflects collective, system‐level self‐efficacy. Confidence was strengthened through coordination, shared learning and organisational support, with *coherence* enhanced by interprofessional alignment.

### 3.2 Affective Attitude and Ethicality

These constructs captured participants emotional and moral responses. Across both professional and caregiver groups, participants expressed optimism about integrating dietitians into primary dental care for children, valuing their specialist nutrition knowledge and behaviour change expertise. Dietitian involvement was viewed as a logical extension of preventive oral‐health promotion and an opportunity to strengthen dietary counselling that dental teams felt sometimes can be time limited.

… dietitians ask a huge range of questions which is so fantastic, and that means that you can really tailor your advice according to that. We don't have A) the time, or B) the knowledge to do that. So to have the option to refer to a dietitian, that would be great. I would totally do it.

(DENT‐10)

However, enthusiasm coexisted with emotional discomfort surrounding weight related discussions and BMI measurement. While some caregivers perceived their child's BMI as healthy, they anticipated experiencing embarrassment, anxiety, hesitation or offence, if assessments indicated their child was living with obesity.

I would be mortified if somebody in front of me said that my child was obese.

(CARE‐6)

Professionals recognised these sensitivities and described the ethical challenge of addressing weight without stigma. Acceptability depended on framing discussions around health, growth and lifestyles rather than weight.

I think there's an element of ‘weight is not what it's all about’… I'm very keen to give the message that it's not about weight, it's about healthiness… I think you've got to come at it from a health perspective rather than a purely weight perspective.

(CARE‐5)

Participants described key safeguards essential for effective weight measurements and discussions, including patient choice, empathy, privacy and sensitive language. Emotional comfort and ethical framing were closely intertwined; enthusiasm for weight‐related aspects of collaboration was sustained only when the process was perceived as respectful, safe and aligned with family values.

Across participant groups, dietitians were perceived as ethically better positioned than dental professionals to manage sensitive issues, due to their expertise in discussing dietary and anthropometric topics with sensitivity.

It needs to be that the dietitian is dealing with that, not the dentists, because the dietitian is trained to deal with that… the dentist aren't.

(CARE‐6)

Affective attitude and ethical dimensions jointly shaped participants´ perceptions of effectiveness and acceptability. There was a stronger preference for dietitians to deliver oral health‐focused dietary advice, while discussions around weight remained ambivalent. Integration of dietitians into care was generally supported when it offered preventive benefits and safeguarded emotional and moral wellbeing.

### 3.3 Burden and Opportunity Costs

This construct reflected perceived amount of effort required to engage in integrated dietetic‐dental care. Participants across all groups acknowledged the effort and time required to engage with an integrated care model. Caregivers described logistical challenges such as missed school and longer appointments if dietetic follow‐ups were added, while dental professionals highlighted the additional workload and coordination required during early implementation.

Despite these short‐term burdens, participants viewed the investment as worthwhile, anticipating longer‐term efficiency and health improvements.

And the benefits [of dietitian involvement] always outweigh because the amount of workload in [the] long run, will reduce… you'll see more patients rather than treating them. So in a long run it will always be beneficial not only to the business but also to the community in general.

(DENT‐2)

The opportunity costs associated with BMI measurement and weight‐related discussions emerged in contexts where existing anxieties about weight or dental settings could discourage families from attending appointments, thereby increasing the risk of poorer oral health outcomes over time.

Some caregivers described prioritising such appointments when they believed it would improve their child's health, while others noted that motivation to attend dietetic appointments might vary between families. Socioeconomic, knowledge and contextual factors could further influence engagement with dietetic advice, with mixed‐perceptions about healthy eating being costly, while some argued it was more time‐consuming and required motivation.

I think they don't they don't understand. They don't listen to education because they're not willing to listen. But two is that, they can't afford erm really like sort of non‐processed healthy organic diet. So I think that's against them.

(DENT‐10)

Overall, the burden was perceived as front‐loaded but balanced by the expectation of long‐term benefits. Opportunity costs centred on perceptions of fairness and effectiveness; integration was considered acceptable when systems demonstrated sensitivity to caregiver motivation and circumstances.

### 3.4 Intervention Coherence

This construct captured participants understanding of the purpose and rationale behind integrated dietetic‐dental care. Participants demonstrated a strong conceptual understanding of the proposed integration, describing it as a logical and complementary fit between diet and oral health. Some extended this reasoning to include weight‐related considerations as well.

The dietitian is, to my mind generally to provide diet advice. So if you're giving diet advice for the body, why not give it for the mouth and the teeth?

(CARE‐3)

This coherence reinforced the perceived legitimacy and relevance of the integration. However, participants identified gaps in current interprofessional communication which, if left unaddressed, could undermine coherence in practice. Both dental professionals and dietitians reflected on experiences where families received conflicting dietary advice, particularly in cases involving restrictive eating or medical complexity.

I think, like, from a patient's point of view, like, who wins? Like who's word trump's the other? Like it's it must be a really difficult position for them to be in…. And it's like we need to agree the priority here.

(DIET‐2)

One caregiver also noted that awareness of dietitian's preventive roles could be limited, which could affect caregiver engagement and motivation. Moreover, three participants (two caregivers, one dental professional) questioned whether BMI measurement was necessary if oral‐health advice already promoted balanced nutrition, a reflection that intertwined with ethical concerns about scope and purpose.

Perceived effectiveness again shaped perceptions: integration was considered coherent and worthwhile when its purpose was clearly communicated, professional roles were delineated and messaging remained consistent across professionals.

### 3.5 Self‐Efficacy

While intervention coherence reflected participants' understanding of the purpose and value of integrated care, self‐efficacy captured professionals' individual and collective confidence, in their ability to implement and sustain integrated working. Professional participants described confidence in delivering integrated care as a shared and relational capacity rather than an individual trait. Successful collaboration was seen to depend on mutual trust, communication and system‐level readiness, including clear referral pathways, well‐defined roles and managerial support.

I think it's talking it's communicating and education both ways. Understanding where they're… you trying to understand where they're coming from… So actually, if you understand where they're coming from and what they're trying to get. And they can understand where you're coming from. There will be a mutual ground and a mutual respect.

(DIET‐3)

Both professions recognised existing fragmentation in interprofessional working, with limited communication and differing scopes of practice, occasionally leading to conflicting advice. Three dietitians reported instances where dental teams had contacted them via phone or letter, while in other cases information about dietary advice was relayed indirectly through patients. Dietitians and dental professionals emphasised the need for bi‐directional learning and interprofessional training to enhance understanding and confidence.

They [dental professionals] never contact us directly, it always comes via a third party.

(DIET‐6)

Joint training, shared protocols, streamlined referral processes and dietetic support embedded within local teams were suggested as strategies to address these challenges.

Perceived effectiveness was closely linked to a sense of collective self‐efficacy. Professionals emphasised the importance of competences and support as essential conditions for achieving preventive aims. Acceptability, therefore, depended on alignment between individual capability and organisational structure; participants needed to feel both capable and supported in order to engage in integrative work.

#### 3.5.1 TFA Summary

**Figure 1:**
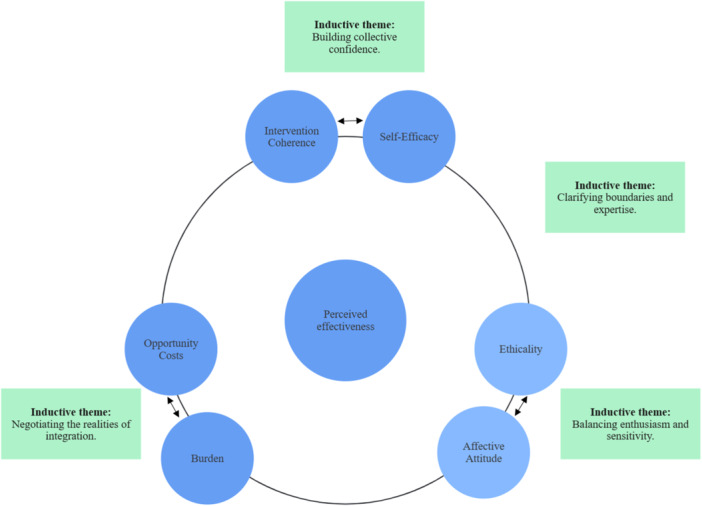
Relationships between inductively generated themes and constructs of the theoretical framework of acceptability (TFA). Blue circles represent TFA constructs, and green boxes represent inductive themes derived from the qualitative analysis. Themes are positioned near the constructs they most strongly informed, with some themes relating to multiple constructs. Arrows indicate conceptual relationships between TFA constructs.

Across constructs, acceptability emerged as a dynamic balance between emotional and ethical alignment, practical feasibility, conceptual clarity and collective confidence. Figure 1 visually depicts the dynamic interaction among these constructs, with perceived effectiveness emerging as the integrative core. Perceived effectiveness functioned as a unifying thread; participants judged certain elements of integration as acceptable if essential safeguards were adopted, ensuring emotional safety, ethically grounding, practical support and organisational viability. Participants evaluated effectiveness in relation to the conditions required for integration to work in practice. For example, dental professionals linked effectiveness to increased opportunities for preventive care through more frequent patient contact, noting:

Patients are more frequent here, so you get to see them more often, whereas at the doctors they only obviously go if they're sick.

(DENT‐5)

The specialist expertise of dietitians was also viewed as central to achieving effective dietary outcomes. However, participants also emphasised that effectiveness would depend on a range of interrelated factors including alignment of professional priorities, appropriate training and organisational support. Perceptions of effectiveness were also shaped by ethical and emotional considerations, including recognition of potential negative outcomes. One caregiver, for example, expressed concern that poorly delivered weight‐related interventions ‘could induce eating disorders’ (CARE‐6), underscoring the importance of sensitive practice. Across stakeholder groups, perceived effectiveness was therefore understood as conditional and relational, emerging from the interaction of emotional and ethical factors. Acceptability was, in turn, shaped by the dynamic interplay between emotional safety, ethical legitimacy and structural support; elements that collectively determine whether integration can be both meaningful and sensitively delivered in practice.

## 4 Discussion

This exploratory study applied the TFA to examine perceptions of dental professionals, dietitians and caregivers regarding the integration of dietitians into primary dental care for children. Integration was conceptualised as co‐location, representing micro‐level integration, with distinct implementation and acceptability considerations compared with other integrated care models, such as warm‐referral pathways and virtual consultations [47]. Overall, acceptability emerged as a relational construct; integration was viewed positively when it felt emotionally safe, ethically legitimate and practically supported. These findings highlight how new preventive roles in healthcare are considered not only in terms of feasibility, but also through their moral and relational fit with the expectations of both families and professionals.

### 4.1 Affective Attitude and Ethicality

Within the TFA, affective attitude and ethicality underpin early judgements of acceptability [38]. Positive attitudes towards dietitian integration co‐existed with emotional sensitivities surrounding weight‐related discussions. While oral health‐focused referrals for dietary support were viewed as ethically straightforward, BMI measurements and feedback often provoked discomfort or anxiety, particularly among caregivers. This ambivalence reflects broader public polarisation in the United Kingdom, where 57.5% of caregivers supported routine height and weight measurements at the dentist, while 27.7% opposed them [22]. Similar reactions are reported in NCMP evaluations, where parents report embarrassment, defensiveness or mistrust of the process, especially when professional feedback conflicted with their own perceptions of their child's body size [48, 49]. Discomfort may also reflect broader scepticism towards government‐led surveillance, reflecting political perceptions, raising questions about the perceived legitimacy of paediatric BMI measurements in public health [49].

Notably, children themselves often view BMI measurements as acceptable and important for their health [50], suggesting discomfort may arise more from how feedback is delivered than from the measurement process itself. Some caregivers in this study reinterpreted clinical terms such as ‘puppy fat’, illustrating the social negotiation of weight labels and the emotional work involved in reconciling professional language with family beliefs [48, 51]. Ethical acceptability, therefore, appeared to depend less on the clinical setting and more on how discussions were framed, communicated and supported.

Nevertheless, for some families, the association between weight measurement and a dental visit may deter attendance, particularly when sensitivities around weight are present. Although existing studies of BMI measurement in dental clinics have not reported such deterrence, this may reflect it was not explicitly assessed [21]. In these contexts, the perceived emotional cost of judgement or embarrassment could outweigh the preventive benefits, underscoring how both the setting and delivery of care shape perceptions of acceptability.

In this study, the focus on BMI measurements to identify weight concerns reflects existing practices in dental settings, albeit with dietitian involvement [21]. While BMI remains an established indicator for identifying overweight and obesity and guiding referral pathways [5], focusing primarily on weight may reduce acceptability. Adopting a more weight‐inclusive approach, emphasising overall health, may better support a sensitive and acceptable model of care. Language played a central role in mediating these dynamics. While banning the term ‘weight’ can create shame and worry [52], caregivers' preferred growth‐ and health‐based framing, highlighting a communication gap. In healthcare settings where emotional sensitivity is already heightened, such as a dental clinic, careful language and framing are particularly critical to maintaining trust. Framing conversations around holistic growth and wellbeing and ensuring clear follow‐up support rather than a one‐off BMI surveillance measurement, may therefore enhance ethical acceptability. These findings underscore how affective attitude and ethicality jointly determine the emotional viability of integration, positioning moral sensitivity as a precondition for perceived effectiveness.

### 4.2 Burden and Opportunity Costs

In the TFA, burden and opportunity costs represent pragmatic determinants of acceptability, reflecting the perceived effort and resources required by an intervention [38]. These factors may need to be balanced against perceptions of the intervention's effectiveness. While dietitian involvement was widely regarded as beneficial for preventive efforts, professionals highlighted practical challenges regarding time and workload. Previous research shows that interprofessional practice can be constrained by limited time for documentation, communication and shared learning, potentially impacting patient outcomes [53]. Similarly, dental professionals anticipated initial increases in administrative and referral burdens, despite potential long‐term benefits including reduced need for restorative treatment. Without adequate structural support, such as clear referral pathways and defined responsibilities, integration could exacerbate rather than alleviate pressures on time‐limited dental teams. However, effective implementation could reduce long‐term dental and systemic health burdens, supporting the shift towards prevention‐focused care models [54, 55]. Caregiver engagement with dietetic services also reflected tensions between burden and opportunity costs. While dietitians were viewed as valuable sources of tailored, evidence‐based dietary support, families facing complex circumstances, competing priorities and financial constraints may have limited engagement. These challenges echo broader research, showing that caregiver motivation, health literacy and logistical barriers can affect healthcare appointment attendance [56], and that competing demands may deprioritise dietary interventions [57]. Misconceptions or limited understanding of dietitians' roles, such as fears that dietary advice would be overly prescriptive, may also act as barriers [58]. This highlights a paradox, what is often perceived as a limitation on the part of families may instead reflect prior experiences of receiving dietary advice in dental care that do not account for wider determinants of dietary behaviour [24, 25]. Expanding awareness of dietitians holistic, behaviour changed focused practice could therefore reduce perceived burden and challenge existing assumptions of dietary advice in dental care.

Although not explored directly in this study, financial frameworks and incentives influence service integration and vary across public and private primary dental care settings. While these factors primarily relate to feasibility, they also shape acceptability through their impact on perceived burden and opportunity costs. In the United Kingdom, integration policy promotes flexible, pooled budgets [59]; however, NHS dental services are commissioned through activity‐based contracts, whereas dietetic services follow separate commissioning pathways [60]. This structural misalignment may hinder integrated working and increase perceived burden by adding coordination demands and limiting continuity across services. In publicly funded settings, commissioning targets prioritising remunerated procedural activity can increase the opportunity costs of allocating clinical time to preventive dietary support. This may constrain opportunities for dietetic integration, despite evidence that dietitians can enhance patient‐centred care, improve service efficiency and deliver cost‐effective interventions in primary care [61, 62]. Conversely, private practices may offer greater flexibility to incorporate dietetic services, potentially reducing organisational burden; however, opportunity costs may be shifted to families, as willingness or ability to pay may introduce inequalities in access. In the United States, dietitians and dental professionals may work within the same federally qualified health centre or integrated healthcare setting, where services are supported through broader medical, dental and federal health‐centre funding structures [63, 64]. However, evidence describing direct collaboration, referral pathways or dedicated funding models specifically between dietitians and dental teams remains limited. One dental professional in our study noted that preventive advice may be less valued than procedural care in private dentistry. While the availability of private paediatric dietetic services suggests some families are willing to pay for specialised dietetic care, acceptability is shaped not only by professional and caregiver attitudes but also by the structural and financial context that determines how burden and opportunity costs are distributed.

### 4.3 Intervention Coherence

Intervention coherence, as a cognitive construct, reflects not just understanding but alignment of professional logic. Participants consistently recognised the interdependence of diet and oral health, viewing integration as a logical extension of preventive care. Three participants noted dietary advice for oral health could also incidentally support weight management, thereby questioning the necessity of explicit BMI measurement. This reflects the overlap between *Delivering Better Oral Health* guidelines and national public health nutrition guidance, both of which promote balanced diets and reduced sugar intake [26]. However, it also has implications for implementation, as it may reduce the perceived need for additional assessment or referral pathways.

Framing identification of concerns solely around BMI or oral health indicators risks privileging more visible or measurable concerns. These conditions do not consistently co‐occur, meaning single‐indicator approaches risk under‐identification. BMI‐based approaches may identify children with unhealthy BMI while missing those with poor dietary quality or dietary support needs despite a healthy‐BMI range. Equally, oral health indicators alone may overlook children with BMI‐related concerns but no evident dental disease. These findings suggest that despite diet being a common risk factor, more flexible and sensitive identification approaches may be warranted, potentially moving beyond single‐indicator referral models towards a more universal approach.

Although this study emphasised the association between BMI and dental caries, our findings suggest that a weight‐centred approach may not be the most acceptable in this context unless essential safeguards are in place. Instead, our data points toward the value of a more health‐centred, behaviour‐focused approach that emphasises sensitivity and overall wellbeing. This aligns with a position statement advocating for health‐focused rather than weight‐focused conversations, which is associated with greater psychosocial wellbeing and fewer unhealthy weight control behaviours [65]. Within this perspective, dietitians could play a meaningful role in supporting both oral and general health through nutrition education, irrespective of a patient's weight status.

Beyond conceptual alignment, professional participants discussed practical coherence, the extent to which integration could operate within existing professional systems. Current practice was characterised by fragmented communication and limited referral pathways, consistent with evidence on professional silos and hierarchical structures constraining collaboration [66, 67, 68]. Competing priorities such as balancing caries prevention with broader nutritional needs, can lead to conflicting dietary advice, particularly in complex clinical situations where eating patterns are limited or nutritionally inadequate. These inconsistencies can compromise patient safety, undermine professional confidence and erode caregiver trust [69]. Without clear mechanisms to reconcile differing priorities, attempts at integration risk reproducing existing divisions under a new guise. This underscores the need for coordinated communication pathways and shared protocols between professions to ensure consistent, holistic and patient‐centred care.

To achieve coherence, participants emphasised the need to cultivate collective models of care, supported by joint training, interprofessional dialogue and mutual respect. This aligns with evidence that collaborative education can improve role clarity, foster professional understanding, reduce resistance to new models of care and promote consistent patient‐centred outcomes [69, 70]. Achieving intervention coherence was therefore understood not as a one‐off consensus but as a dynamic, reflexive process requiring both structural facilitation and relational investment. Within the TFA, intervention coherence provides the cognitive foundation for self‐efficacy, only when professionals share a clear, consistent understanding of each other's roles, can confidence develop in integrated care.

#### 4.3.1 Self‐Efficacy

Professional participants conceptualised self‐efficacy not as an individual trait but as a collective capacity emerging from trust, leadership and system design. Professional confidence to deliver integrated care depended on coherent referral pathways, shared understanding of professional roles and local dietetic support; conditions extending beyond personal competence. This aligns with literature indicating that interprofessional confidence can be shaped by organisational culture and relational climate rather than technical training alone [71].

Several professional participants proposed that joint case discussions, shared learning sessions and dedicated time for peer consultation could strengthen mutual trust and role clarity, resonating with evidence that collaborative practice develops effectively, when teams have opportunities for reciprocal learning and reflection [72, 73]. In this sense, self‐efficacy represents both an individual and collective capability, one that grows through coherent systems, relational trust and visible organisational commitment. This reframing of self‐efficacy as a collective rather than individual capacity aligns with the interprofessional education collaborative core competencies, which emphasises role clarity, respectful communication, shared values and teamwork in delivering coordinated, person‐centred care [74]. Strengthening these elements is likely to enhance both professional assurance and caregivers' confidence in the acceptability and credibility of integrated dietetic‐dental care.

## 5 Limitations

A key limitation is that dental professionals and caregivers were purposively sampled from primary dental care settings in South West England where dental students are trained, which does not reflect typical primary care dental settings across the United Kingdom. However, this setting serves socioeconomically deprived areas, with disproportionately higher rates of obesity and tooth decay, ensuring inclusion of communities most affected by these public health challenges [75]. In contrast, paediatric dietitians were recruited nationwide via convenience sampling, ensuring geographic diversity but creating an imbalance in the diversity of perspectives between professional groups. Future research should recruit dental professionals from more diverse geographic and organisational contexts, including secondary care, where greater interdisciplinary collaboration may shape different perspectives and improve generalisability.

The predominantly White ethnicity of caregivers limits the transferability of the findings to more ethnically diverse populations [76], particularly given higher rates of childhood obesity, dental caries and disparities in dental service access among some minoritised groups [77, 78]. Cultural differences in attitudes towards weight, health monitoring and healthcare engagement may also influence the acceptability of integrated care [79]. Additionally, although caregivers could draw on experiences across age groups, adolescents (13–18 years) may have distinct considerations, including autonomy and sensitivity to weight‐related discussions, which were not explicitly explored. All dietitians were female; while reflective of the gender distribution within the profession, it limits exploration of gender dynamics in interprofessional working. Future research should include more diverse participant groups to better understand how perceptions of integrated dietetic–dental care vary across different demographic and cultural contexts.

Self‐selection bias is also a potential concern, as individuals with a pre‐existing interest in oral health and nutrition may be overrepresented. All caregivers had access to dental care, therefore excluding perspectives of the growing population unable to access public dental services, for whom dietitian integration may seem less important than securing basic oral healthcare. Nonetheless, the study's strength lies in its triangulation of perspectives, offering a multi‐stakeholder view. While member checking was conducted, the low engagement and uniformly affirming feedback limited its contribution to confirmability.

As all interviews were conducted by a single researcher (a dietitian), the shared professional identity with dietitian participants may have subtly influenced interview dynamics. Economic considerations were also not explored in depth during interviews. Participants were not asked specifically about willingness to pay for dietetic input in private dental settings, nor about NHS funding priorities or commissioning models. As financial and commissioning structures are likely to influence both feasibility and acceptability, this represents an important area for future research. Finally, as interviews were guided by hypothetical scenarios, responses may differ from those in real‐world clinical settings. While the study identifies conditions for acceptability, future pilot and feasibility studies are needed to assess how these translate into practice, where factors such as child behaviour, family engagement and organisational constraints may influence implementation.

## 6 Conclusion

This study showed that acceptability was influenced by interrelated emotional, ethical and structural dimensions. Affective attitude and ethicality shaped how participants viewed weight‐related discussions; generally acceptable when framed sensitively and supported by appropriate safeguards. Burden and opportunity costs reflected the practical and motivational effort required of both families and professionals and were deemed more acceptable when integration is supported by clear care pathways and preventive value. Intervention coherence and self‐efficacy were mutually reinforcing; a shared understanding of roles and goals could enable professional confidence and caregiver trust, contingent on system design and structural support. Overall, acceptability was dynamic and dependent on several factors. Differential organisational capacity between public and privately funded clinics raises possible commissioning and equity limitations. Embedding ethical sensitivity, collective capability and structural support will be critical to translating acceptability into sustainable, equitable practice. Future work should examine how these insights can be translated into practical care pathways, and identify the core components required to strengthen collaboration, including interprofessional training opportunities, improved communication systems and supporting organisational structures.

## Author Contributions

**Lauren Hallewell:** conceptualisation, methodology, investigation, formal analysis, data curation, writing – original draft, writing – review and editing, visualisation, validation, project administration. **Raul Bescos:** conceptualisation, methodology, investigation, formal analysis, writing – review and editing, visualisation, validation, supervision, project administration. **Zoe Brookes:** conceptualisation, methodology, investigation, writing – review and editing, visualisation, validation, supervision, project administration. **Tracey Parkin:** conceptualisation, methodology, investigation, writing – review and editing, visualisation, validation. **Robert Witton:** conceptualisation, methodology, investigation, writing – review and editing, visualisation, validation, supervision, project administration. **Patricia Casas‐Agustench:** conceptualisation, methodology, investigation, formal analysis, data curation, writing – original draft, writing – review and editing, visualisation, validation, supervision, project administration.

## Funding

The authors have nothing to report.

## Ethics Statement

The study was approved by University of Plymouth Faculty of Health Research Ethics Committee (Ref: 5706, 5479, 4833).

## Conflicts of Interest

The authors declare no conflicts of interest. Relevant professional affiliations include the British Dietetic Association, British Dental Association, College of Dietitians‐Nutritionists of Catalonia and Member of the Association for Nutritionists.

## Supporting information


Supporting File

## Data Availability

The data that support the findings of this study are available on request from the corresponding author. The data are not publicly available due to privacy or ethical restrictions.
