# Supplementary material for: Exploring the Acceptability of Integrating Dietitians Into Primary Care Dental Services for Children
Source: Health Expect. 2026 Jul 3;29(4):e70753. doi: 10.1111/hex.70753 (PMC13332333; doi:10.1111/hex.70753)
Supplement: Supplementary file 1 — Supporting File [file HEX-29-e70753-s001.docx]

**Online Supplementary Materials**

**Supplementary File 1 (File S1). Interview guides.**

- **1.1. Dental professionals interview guide**
- **1.2. Dietitians interview guide**
- **1.3. Caregivers interview guide**
- **1.4. Background information provided in dietitians interviews**
- **1.5. Hypothetical scenarios**

**Supplementary Table 1 (Table S1). Demographics of dental professionals interviewed.**

**Supplementary Table 2 (Table S2). Demographics of dietitians interviewed.**

**Supplementary Table 3 (Table S3). Demographics of caregivers interviewed.**

**Supplementary File 1. Interview guides.**

Note: Minor rewording was made to improve clarity and professional tone for publication. These changes did not alter the core themes, intent, or ethical sensitivity of the questions or scenarios originally approved by the ethics committee.

**1.1 Dental professional interview guide**

**Weight Management Experience**

- Have you ever measured the height and weight of paediatric patients in a dental setting?

If so, what prompted you to do this, and what was your experience like?

- Have you ever undertaken any activities related to weight management for paediatric patients?
- How receptive are you as a dental professional to discussing paediatric patients’ weight at the dental clinic? Please explain why.

**Dietary Assessment and Advice Experience**

- What kind of dietary advice do you currently provide to paediatric patients?
- Do you follow any specific guidelines? If so, which ones?
- Do you set dietary goals with paediatric patients? If yes, what approaches or techniques do you use?
- Do you think the dietary advice you provide is successful in improving the oral health of your patients? If yes, why is that? If no, what needs to change?

**Experience with Dietitians**

- Have you ever referred a patient to a dietitian? If so, what were the reasons for referral, and what was your experience like?
- Do you think dietitians can promote good oral health through providing dietary advice? If yes, what would this look like in practice? If no, why not?

**Reactions to Dietitian Role Video**

(Participants will watch/listen to a video produced by the British Dietetic Association: <https://www.youtube.com/watch?v=4RmLhe72Htc>)

- Do you have any questions about the role of a dietitian after watching this?

**Hypothetical Scenario 1: Dietitian Screening for BMI in a Dental Setting**

(Interviewer reads a scenario about a dietitian measuring children's BMI in a dental clinic and offering referrals to an in-house dietetic clinic.)

- Do you have any questions about the scenario?
- Do you think it is appropriate for a dietitian to work within the dental setting to screen for paediatric patient’s body weight and to provide dietary advice aimed at addressing both oral health and body weight? If yes, why? If no, why not?
- To what extent do you think referring a paediatric patient who are underweight, overweight or obese to an in-house dietitian could improve their oral health? In what ways?
- To what extent do you think such referrals could help improve the body weight of your paediatric patients? Please explain why.
- If dietitians were to screen children’s body weight and offer referrals in your practice, what changes do you think this might bring to your dental clinic?

**Hypothetical Scenario 2: Dietitian Support for Implementing Dietary Goals Set by Dentists**

(Interviewer reads a scenario about a dentist setting dietary goals for a child with high levels of tooth decay and referring them to a dietitian for support.)

- Do you have any questions about the scenario?
- If you referred patients to an in-house dietitian for support in implementing your dietary advice, do you think this would impact your staff workload? And in what way?
- To what extent (if any) do you think referring patients with diet-related oral health problems (such as tooth decay) to a dietitian could help improve their oral health? In what ways?
- If you were to refer a patient to an in-house dietitian to help implement your dietary advice for diet-related oral health issues, what changes do you think might occur in your dental consultation?
- How do you view the potential benefits and risks of involving dietitians to support families in implementing your dietary advice? Do you feel one might outweigh the other?

**Barriers and Facilitators to Referral to a Dietitian in the Dental Clinic**

- Can you think any challenges, if any, to working interprofessionally with dietitians?
- Can you think of any differences, if any, in dietary advice provision between dietitians and dentists? If so, do you think this could affect the dental clinic and your patient’s oral health?
- By referring to your patients to a dietitian for dietary support, do you think there would be any changes to your staff workload?
- Can you explain if there is anything that would restrict you from making a referral to a dietitian? What are they and why?
- Can you explain if there is anything that would encourage you to refer a patient to the dietitian? What are they and why?
- How do you think it would be best to overcome these restrictions?
- Are there any other thoughts/comments you would like to make on this topic?

**1.2 Dietitian interview guide**

**Oral Health Experience**

- Have you ever assessed the oral health of a paediatric patient? If yes, in what context?
- Have you encountered situations where advice from a dental professional influenced your dietetic management plan? If so, what was your experience?
- Have you ever had to adjust a dietetic management plan based on a child’s oral health? If so, what was your experience?
- Have you ever encountered patients with pre-existing beliefs about foods/drinks, etc. that can impact oral health? If yes, what were these beliefs, and how did you manage this?
- Have you ever communicated with a dental professional about a shared patient? If so, in what capacity?

**Oral Health Dietary Advice Experience**

- Do you provide oral health-related dietary advice to paediatric patients? If yes, what does this involve? Do you follow specific guidelines? If so, which ones?
- Do you set oral health-related dietary goals with your paediatric patients? If yes, do you have any techniques that assist with this?
- Do you think the dietary advice you provide is successful in improving the oral health of your patients? If yes, why is that? If no, what needs to change?

**Background Information on Diet Advice and BMI Screening in Dental Settings**

(The interviewer will provide a short overview of research on the relationship between BMI and dental caries, dietary interventions in dental settings, and challenges reported by dental professionals regarding healthy weight interventions).

**Reactions to Hypothetical Scenario 1: BMI Screening by a Dietitian in a Dental Clinic**

(The interviewer presents a scenario where a dietitian screens children's BMI in a dental setting and offers referrals for nutrition support).

- Do you have any questions about this scenario?
- Do you think it is appropriate for a dietitian to utilise the dental setting to screen paediatric patients for body weight and to provide dietary advice for both oral health and body weight? If yes, why? If no, why not?
- To what extent do you think a referral to a dietitian could assist in improving the oral health of underweight/overweight/obese paediatric patients? In what way?
- To what extent do you think a referral to a dietitian could assist improving the body weight of paediatric patients? Please explain why.
- By having a dietitian screen for body weight and providing paediatric patients with the option for referral in a dental setting, what changes do you think may occur in their dental experience?

**Reactions to Hypothetical Scenario 2: Dietitian Supporting Dietary Goals Set by a Dentist**

(The interviewer presents a scenario where a dental professional sets dietary goals for a child with high levels of tooth decay and refers them to a dietitian for further support).

- Do you have any questions about this scenario?
- To what extent do you think a referral to a dietitian could assist in improving the oral health of patients that experience diet-related oral health problems like tooth decay? In what way?
- How confident would you feel in supporting families in implementing dietary goals set by a dental professional?
- Are there any factors that would act as a barrier to you integrating in the dental clinic in this scenario? If so, what is it and please expand?
- Are there any factors that would act as a facilitator to you supporting the dental clinic in this scenario? If so, what is it and please expand.

**Barriers and Facilitators to Interprofessional Collaboration with Dental Professionals**

- Can you think of any challenges, if any, to working interprofessionally with dental professionals?
- Can you think of any differences in the dietary advice provided by dietitians and dental professionals?
- How do you think we could foster a more consistent or harmonised approach to the dietary advice you provide to patients?
- Is there anything you feel you would need in order to support your role as a dietitian working within a dental setting?
- Do you have any other thoughts or comments on this topic?

**1.3 Caregiver interview guide**

**Weight and Height Measurement in Dental Settings**

- Has your child ever had their weight and height measured at the dentist? If so, did the dental professional explain the reason, and how did you find the experience?
- How comfortable are you, as a caregiver, with discussing your child’s weight during a dental visit? Please explain why.

**Dietary Assessment and Advice in Dental Settings**

- Have you ever completed a dietary assessment with a dental professional (on behalf or with your child)? Including verbal discussions about your child’s dietary intake or being asked to complete a food diary? If so, what was your experience of this?
- Has your child ever received dietary advice from a dental professional, regarding oral health or body weight? If so, what was advised and what was your experience?
- Has a dental professional ever set a dietary goal with your child regarding oral health or body weight? If so, did you feel it was achievable and did you feel supported in how to achieve the goal?

**Experience with Dietitians**

- Has your child ever been referred to a dietitian (beyond weight management)? If so, what was your experience like?
- Do you think dietitians can promote good oral health through providing dietary advice? If yes, what does that look like if no, why is that?

**Reactions to Dietitian Role Video**

(Participants will watch/listen to a video produced by the British Dietetic Association: <https://www.youtube.com/watch?v=4RmLhe72H>)

- Do you have any questions about the role of a dietitian after watching this?

**Dietitians Conducting BMI Screening and Providing Weight Management Support- First hypothetical scenario read out**

- Do you have any questions on the scenario?
- Do you think it is appropriate for a dietitian to be using the dental setting to screen for children’s body weight and to provide dietary advice to address oral health and body weight? If yes, why is that? If no, why is that?
- To what extent do you think a referral to a dietitian could assist improving the oral health of underweight/overweight/obese children? In what way?
- To what extent do you think a referral to a dietitian could assist improving the body weight of children?
- By having the option to screen for body weight and referral to a dietitian, what, if any, changes do you think may occur to your dental experience?

**Referrals to a Dietitian for Oral Health Issues- Second Hypothetical Scenario Read Out**

- Do you have any questions on the scenario?
- Do you think it is appropriate for a dentist to be referring to a child to a dietitian for support in implementing dietary advice, that has been set by the dentist? If yes, why is that? If no, why is that?
- To what extent do you think a referral to a dietitian could assist improving the oral health of patients that experience diet-related oral health problems like tooth decay? In what way?
- By having the option for a dentist to make a referral to a dietitian due to diet related oral health problems, what changes, if any, do you think may occur to your dental experience?

**Barriers and Facilitators to Attending a Dietetic Appointment**

- Can you explain if there is anything that would restrict you and your child from attending a referral to a dietitian, to discuss oral health or body weight?
- How do you think it would be best to overcome these restrictions?
- Is there anything that would encourage you and your child to attend a referral to a dietitian to discuss oral health or body weight? What is it and why?
- Is there anything that would restrict you from making dietary changes with your child, as recommended by a dietitian and dentists? If so, what support would you need to overcome these restrictions?
- Are there any other thoughts/comments you would like to make on this topic?

**1.4 Background information provided in dietitians interviews**

The UK Public Health report on BMI and dental caries showed underweight, overweight and very overweight children were more likely to have experienced dental caries than those of a healthy weight (1). Dietary assessment and advice in the dental clinic can play a crucial role for preventing and managing poor oral health and obesity (2). Dental clinics, given their frequent contact with patients (3), could be an appropriate location to support dietary behaviour change and BMI screening (4,5). A recent study by Large *et al.,* in Scotland highlights that BMI screening conducted by dental professionals was widely accepted by caregivers and children attending the dental setting (6). However, dental professionals report that engaging in healthy weight interventions with caregivers and children can be challenging (7). Key barriers include fear of offending patients when communicating results, lack of knowledge and training on BMI calculations and counselling skills, time constraints, lack of referral pathways, and insufficient reimbursement (4, 8-12).

Research suggests the oral health dietary advice in the dental setting, is of variable quality, brief, or sometimes given with minimal patient interaction (13-15). Several factors such as socio-economic status, knowledge, cooking facilities/skills and motivation to change, which can influence the success of changing dietary behaviours, is not generally explored or considered by the dental professional when setting a dietary goal (16). Similarly, dental professionals report time constraints, personal perceptions of nutrition importance, lack of insurance reimbursements, insufficient training, and low confidence as barriers to providing oral health dietary advice (17-19). Furthermore, generally dental professionals will explain the findings of their clinical examinations and the significance of taking particular actions when providing dietary advice (20), which tends to be focused on sugar consumption and frequency (‘sugar hits') (16), alongside fluoride and toothbrushing advice (2).

*References for background information provided in dietitians interviews*

1. Public Health England. *The relationship between dental caries and body mass index: Child level analysis.* London: Public Health England.

2. GOV.UK. *Delivering better oral health: an evidence-based toolkit for prevention*. 2021 [cited 2024 Jan 2]. Available from: <https://www.gov.uk/government/publications/delivering-better-oral-health-an-evidence-based-toolkit-for-prevention>

3. Harris R, Gamboa A, Dailey Y, Ashcroft A. One-to-one dietary interventions undertaken in a dental setting to change dietary behaviour. *Cochrane Database Syst Rev*. 2012;2012(3):CD006540.

4. Curran AE, Caplan DJ, Lee JY, Paynter L, Gizlice Z, Champagne C, et al. Dentists’ attitudes about their role in addressing obesity in patients: a national survey. *J Am Dent Assoc*. 2010;141(11):1307–16.

5. Large JF, Madigan C, Graham H, Biddle GJH, Sanders J, Daley AJ. Public and dental teams’ views about weight management interventions in dental health settings: Systematic review and meta-analysis. *Obes Rev*. 2024;e13726.

6. Daley AJ. Time to get our teeth into reducing obesity: should dentists screen and deliver interventions to reduce obesity in the population? *Br Dent J*. 2022;232(2):78–9.

7. Ames H, Mosdøl A, Blaasvær N, Nøkleby H, Berg RC, Langøien LJ. Communication of children’s weight status: what is effective and what are the children’s and parents’ experiences and preferences? A mixed methods systematic review. *BMC Public Health*. 2020;20(1):574.

8. Lee JY, Caplan DJ, Gizlice Z, Ammerman A, Agans R, Curran AE. US pediatric dentists’ counseling practices in addressing childhood obesity. *Pediatr Dent*. 2012;34(3):245–50.

9. Tseng R, Vann WF, Perrin EM. Addressing Childhood Overweight and Obesity in the Dental Office: Rationale and Practical Guidelines. *Pediatr Dent*. 2010;32(5):417–23.

10. Braithwaite AS, Vann WF, Switzer BR, Boyd KL, Lee JY. Nutritional counseling practices: how do North Carolina pediatric dentists weigh in? *Pediatr Dent*. 2008;30(6):488–95.

11. Cole DDM, Boyd LD, Vineyard J, Giblin-Scanlon LJ. Childhood Obesity: Dental hygienists’ beliefs attitudes and barriers to patient education. *J Dent Hyg*. 2018;92(2):38–49.

12. Tavares M, Chomitz V. A healthy weight intervention for children in a dental setting: a pilot study. *J Am Dent Assoc.* 2009;140(3):313–6.

13. Moynihan PJ. Dietary advice in dental practice. *Br Dent J*. 2002;193(10):563–8.

14. Arora A, Lam AS, Karami Z, Do LG, Harris MF. How readable are Australian paediatric oral health education materials? *BMC Oral Health.* 2014;14(1):111.

15. Watt R, Mcglone P, Kay E. Prevention. Part 2: Dietary advice in the dental surgery. *Br Dent J*. 2003;195:27–31.

16. Franki J, Hayes MJ, Taylor JA. The provision of dietary advice by dental practitioners: a review of the literature. *Community Dent Health.* 2014;31(1):9–14.

17. Hayes MJ, Wallace JP, Coxon A. Attitudes and barriers to providing dietary advice: perceptions of dental hygienists and oral health therapists. *Int J Dent Hyg*. 2016;14(4):255–60.

18. Levy TA, Raab CA. A study of the dietary counseling practices among Oregon dental hygienists. *J Dent Hyg*. 1993;67(2):93–100.

19. McKinney L, Karp NV, Karp WB. Dentist practices and attitudes toward nutrition counseling. *J Mass Dent Soc*. 1996;44(4):10–3.

20. Newton JT, Asimakopoulou K. Minimally invasive dentistry: Enhancing oral health related behaviour through behaviour change techniques. *Br Dent J.* 2017;223(3):147–50.

**1.5 Hypothetical scenarios**

Scenario 1:

The dental setting may play a valuable role in the early detection of underweight, overweight, and obesity by measuring children's height and weight to calculate Body Mass Index (BMI). Compared to other healthcare environments such as GP surgeries or hospitals, dental clinics typically have more frequent contact with paediatric patients, presenting an opportunity for broader health screening.

Lisa is a 10-year-old patient attending the dental clinic for a routine appointment, accompanied by her caregiver, David. While in the dental waiting room, they are informed that children attending the clinic have the option of undergoing BMI screening to identify and provide necessary support. Screening is available to all patients, regardless of weight status.

David consents for a registered dietitian to measure Lisa’s height and weight in a private room, prior to her dental appointment. After calculating Lisa’s BMI, the dietitian asks David whether he would like Lisa to be present for the discussion of the results, to which he agrees. The dietitian then sensitively informs them that Lisa’s BMI falls within the overweight category and offers a referral to a dietetic consultation within the dental setting.

At the consultation, Lisa and David attend a 45-minute consultation with a registered dietitian. During this session, a range of topics are covered, including a dietary assessment, questions regarding general and oral health, medications, lifestyle behaviours, and potentially relevant socioeconomic factors such as access to cooking facilities and food security.

The dietitian explores the family’s readiness to implement dietary changes and addresses any concerns or barriers they may have. Together, the dietitian, Lisa, and David develop specific, measurable, achievable, and time-bound dietary goals tailored to the family’s needs and capabilities. Using behaviour change techniques, the dietitian provides ongoing support and motivation to help implement these goals. Regular 30-minute follow-up appointments are offered to monitor progress and adjust the plan as necessary.

Scenario 2:

Jack is an 8-year-old child patient attending the dental clinic for a routine appointment, accompanied by his caregiver, Molly. During the consultation, the dental professional assesses Jack’s dietary habits in light of significant tooth decay. As part of the consultation, the dental professional works with Jack and Molly to set a dietary goal, which includes reducing all of Jack’s intake of fizzy drinks (currently six per day) and limiting sweets before bedtime.

To support implementation of this goal, the dental professional suggests referring Jack and Molly to a registered dietitian available at the dental clinic. Molly and Jack accept the referral and attend a 45-minute consultation with the dietitian.

The consultation includes a comprehensive dietary assessment as well as discussion of general and oral health, medications, lifestyle behaviours, and socioeconomic factors such as cooking facilities and food security. The dietitian explores the family's readiness to make dietary changes and addresses any concerns or barriers. Working collaboratively with Jack and Molly, the dietitian helps to implement the dietary goals established in the dental appointment, tailoring them to the family’s specific needs and circumstances.

Using behaviour change techniques, the dietitian supports Jack and Molly in making sustainable dietary adjustments. Regular 30-minute follow-up appointments are offered to monitor progress and modify goals as needed.

**Supplementary Table 1. Demographics of dental professionals interviewed.**

| **Demographic variable** | **Categories** | **Frequency (n)** |
| --- | --- | --- |
| **Dental professional role** |  |  |
|  | General Dentist | 3 |
|  | Dental Hygienist | 2 |
|  | Dental Therapist | 2 |
|  | Dental Nurse | 3 |
| **Years of dental experience** |  |  |
|  | 1-10 years |  |
|  | 11-20 years | 6 |
|  | 21-30 years | 2 |
|  | 30 + years | 2 |
| **Work setting (in addition to primary care paediatric dental clinics) *†** |  |  |
|  | Private dentistry | 5 |
|  | Academia (Lecturing/supervision) | 2 |
|  | Community dental clinic | 2 |
|  | Research | 2 |
|  | Outreach work | 1 |
|  |  |  |
| **Gender †** |  |  |
|  | Female | 6 |
|  | Male | 1 |
| **Ethnic Background †** |  |  |
|  | White British | 6 |
|  | Asian/Asian British- Indian | 1 |
| **Age †** |  |  |
|  | Under 25 years | 0 |
|  | 25-34 years | 2 |
|  | 35-44 years | 1 |
|  | 45-54 years | 2 |
|  | 55-64 years | 2 |

† 7 out of 10 participants completed the demographic survey; missing data are due to non-responses

*Participants could select multiple options; thus, the total frequency exceeds the number of respondents (n=7).

**Supplementary Table 2. Demographics of dietitians interviewed.**

| **Demographic variable** | **Categories** | **Frequency (n=6)** |
| --- | --- | --- |
| **Years of dietetic experience** |  |  |
|  | 1-10 years | 2 |
|  | 11-20 years | 1 |
|  | 21-30 years | 2 |
|  | 30 + years | 1 |
| **Geographical Location** |  |  |
|  | South West | 1 |
|  | Yorkshire and the Humber | 1 |
|  | South East | 1 |
|  | West midlands | 1 |
|  | East midlands | 1 |
|  | North West | 1 |
| **Work setting** |  |  |
|  | District General Hospital | 2 |
|  | Community | 3 |
|  | Acute with outpatient appointments | 1 |
| **Private/NHS practice** |  |  |
|  | NHS | 5 |
|  | NHS with prior private experience | 1 |
| **Gender** |  |  |
|  | Female | 6 |
| **Ethnic Background** |  |  |
|  | White British | 6 |
| **Age** |  |  |
|  | Under 25 years | 1 |
|  | 25-34 years | 0 |
|  | 35-44 years | 1 |
|  | 45-54 years | 2 |
|  | 55-64 years | 2 |

**Supplementary Table 3. Demographics of caregivers interviewed.**

| **Demographic variable** | **Categories** | **Frequency (n) †** |
| --- | --- | --- |
| **Highest level of education** |  |  |
|  | Secondary school | 1 |
|  | Vocational training | 1 |
|  | Bachelor’s degree | 2 |
|  | Master’s degree | 1 |
| **Employment status** |  |  |
|  | Employed full-time | 2 |
|  | Employed part time | 1 |
|  | Self-employed | 1 |
|  | Homemaker | 1 |
| **Gender** |  |  |
|  | Female | 4 |
|  | Male | 1 |
| **Relationship with the child/ren discussed in the interview** |  |  |
|  | Mother | 4 |
|  | Father | 1 |
| **Number of children caring for** |  |  |
|  | 2 children | 3 |
|  | 3 children | 2 |
| **Age ranges of children caring for *** |  |  |
|  | 3-5 years | 1 |
|  | 6-8 years | 0 |
|  | 9-11 years | 5 |
|  | 12-14 years | 5 |
| **Ethnic background** |  |  |
|  | White British | 3 |
|  | White Irish | 1 |
|  | Asian/Asian British- Chinese | 1 |
| **Age** |  |  |
|  | Under 25 years | 0 |
|  | 25-34 years | 0 |
|  | 35-44 years | 2 |
|  | 45-54 years | 3 |
|  | 55-64 years | 0 |

† 5 out of 7 participants completed the demographic survey; missing data are due to non-responses

*Participants could select multiple options; thus, the total frequency exceeds the number of respondents (n=5).
